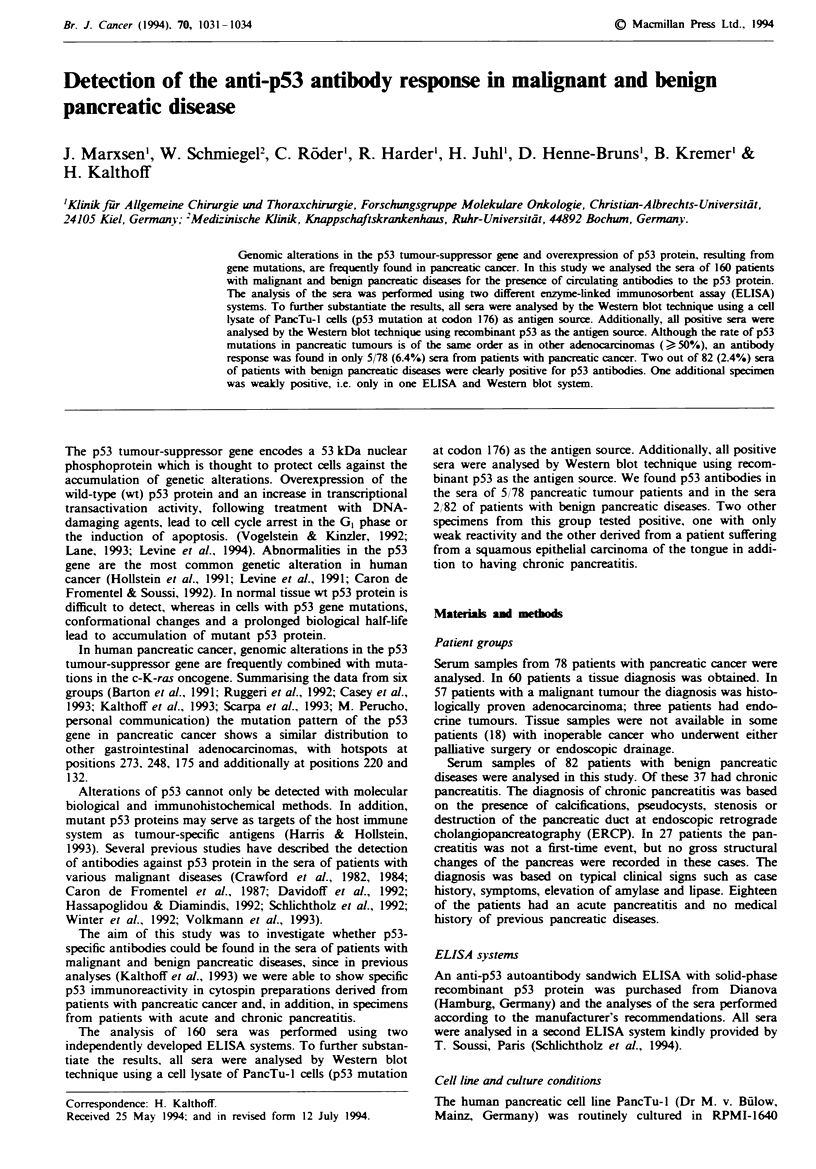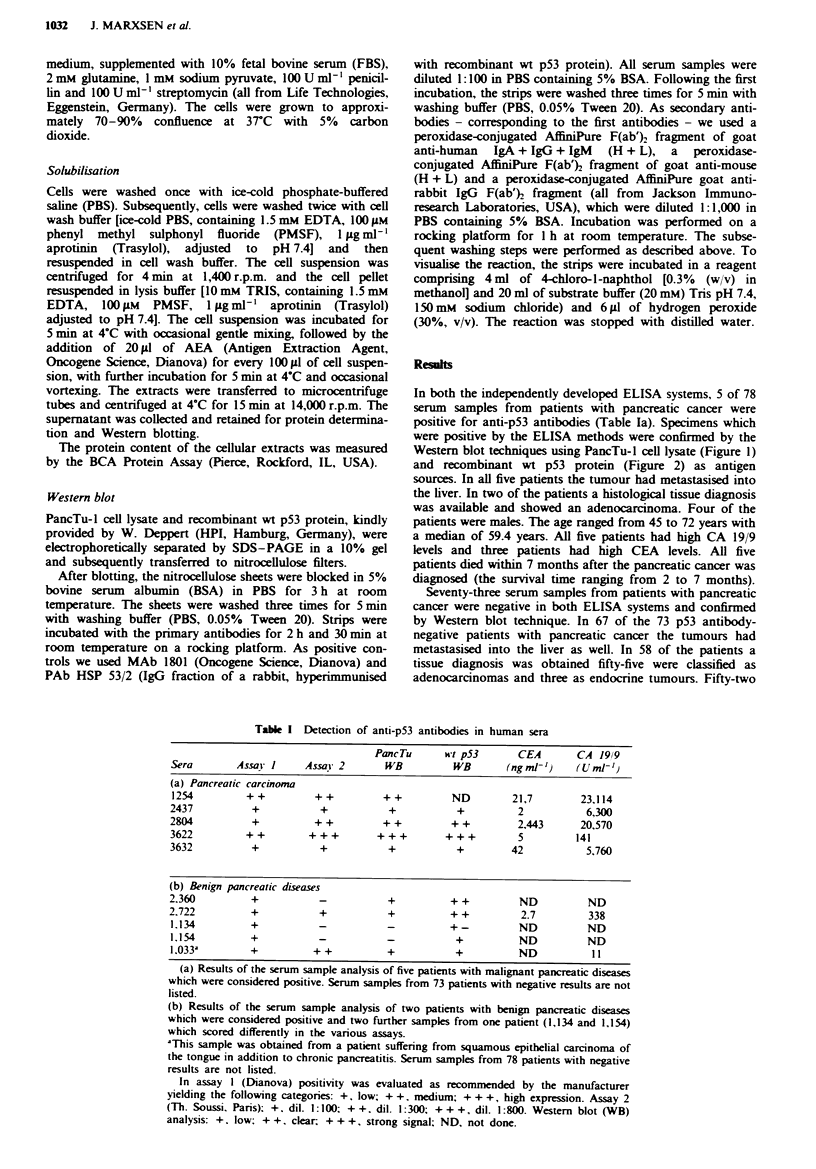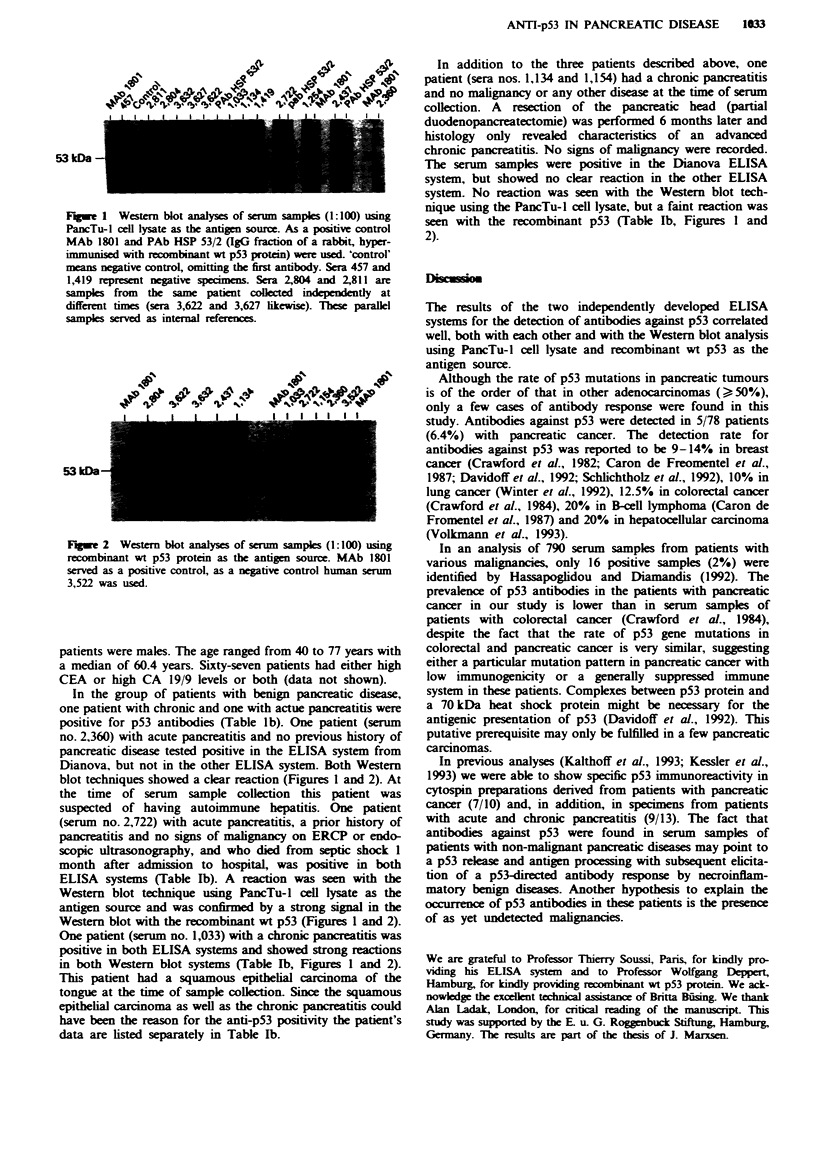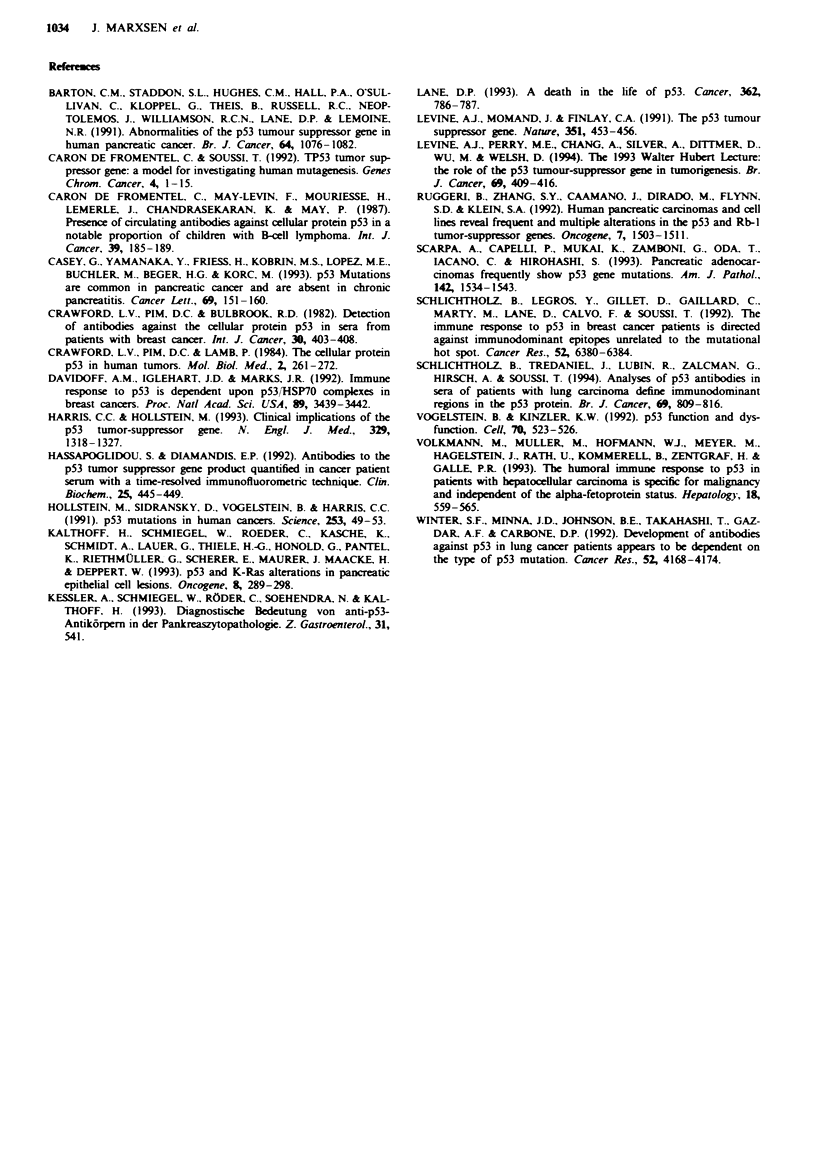# Detection of the anti-p53 antibody response in malignant and benign pancreatic disease.

**DOI:** 10.1038/bjc.1994.443

**Published:** 1994-11

**Authors:** J. Marxsen, W. Schmiegel, C. Röder, R. Harder, H. Juhl, D. Henne-Bruns, B. Kremer, H. Kalthoff

**Affiliations:** Klinik für Allgemeine Chirurgie und Thoraxchirurgie, Forschungsgruppe Molekulare Onkologie, Christian-Albrechts-Universität, Kiel, Germany.

## Abstract

**Images:**


*Br. J. Cancer* (1994), **70**, 1031–1034 © Macmillan Press Ltd., 1994

### Detection of the anti-p53 antibody response in malignant and benign pancreatic disease

J. Marxsen[1], W. Schmiegel[2], C. Röder[1], R. Harder[1], H. Juhl[1], D. Henne-Bruns[1], B. Kremer[1] & H. Kalthoff

[1]*Klinik für Allgemeine Chirurgie und Thoraxchirurgie, Forschungsgruppe Molekulare Onkologie, Christian-Albrechts-Universität, 24105 Kiel, Germany;* [2]*Medizinische Klinik, Knappschaftskrankenhaus, Ruhr-Universität, 44892 Bochum, Germany.*

Genomic alterations in the p53 tumour-suppressor gene and overexpression of p53 protein, resulting from gene mutations, are frequently found in pancreatic cancer. In this study we analysed the sera of 160 patients with malignant and benign pancreatic diseases for the presence of circulating antibodies to the p53 protein. The analysis of the sera was performed using two different enzyme-linked immunosorbent assay (ELISA) systems. To further substantiate the results, all sera were analysed by the Western blot technique using a cell lysate of PancTu-1 cells (p53 mutation at codon 176) as antigen source. Additionally, all positive sera were analysed by the Western blot technique using recombinant p53 as the antigen source. Although the rate of p53 mutations in pancreatic tumours is of the same order as in other adenocarcinomas ($\geqslant$50%), an antibody response was found in only 5/78 (6.4%) sera from patients with pancreatic cancer. Two out of 82 (2.4%) sera of patients with benign pancreatic diseases were clearly positive for p53 antibodies. One additional specimen was weakly positive, i.e. only in one ELISA and Western blot system.

The p53 tumour-suppressor gene encodes a 53 kDa nuclear phosphoprotein which is thought to protect cells against the accumulation of genetic alterations. Overexpression of the wild-type (wt) p53 protein and an increase in transcriptional transactivation activity, following treatment with DNA-damaging agents, lead to cell cycle arrest in the $G_1$ phase or the induction of apoptosis. (Vogelstein & Kinzler, 1992; Lane, 1993; Levine *et al.*, 1994). Abnormalities in the p53 gene are the most common genetic alteration in human cancer (Hollstein *et al.*, 1991; Levine *et al.*, 1991; Caron de Fromentel & Soussi, 1992). In normal tissue wt p53 protein is difficult to detect, whereas in cells with p53 gene mutations, conformational changes and a prolonged biological half-life lead to accumulation of mutant p53 protein.

In human pancreatic cancer, genomic alterations in the p53 tumour-suppressor gene are frequently combined with mutations in the c-K-*ras* oncogene. Summarising the data from six groups (Barton *et al.*, 1991; Ruggeri *et al.*, 1992; Casey *et al.*, 1993; Kalthoff *et al.*, 1993; Scarpa *et al.*, 1993; M. Perucho, personal communication) the mutation pattern of the p53 gene in pancreatic cancer shows a similar distribution to other gastrointestinal adenocarcinomas, with hotspots at positions 273, 248, 175 and additionally at positions 220 and 132.

Alterations of p53 cannot only be detected with molecular biological and immunohistochemical methods. In addition, mutant p53 proteins may serve as targets of the host immune system as tumour-specific antigens (Harris & Hollstein, 1993). Several previous studies have described the detection of antibodies against p53 protein in the sera of patients with various malignant diseases (Crawford *et al.*, 1982, 1984; Caron de Fromentel *et al.*, 1987; Davidoff *et al.*, 1992; Hassapoglidou & Diamindis, 1992; Schlichtholz *et al.*, 1992; Winter *et al.*, 1992; Volkmann *et al.*, 1993).

The aim of this study was to investigate whether p53-specific antibodies could be found in the sera of patients with malignant and benign pancreatic diseases, since in previous analyses (Kalthoff *et al.*, 1993) we were able to show specific p53 immunoreactivity in cytospin preparations derived from patients with pancreatic cancer and, in addition, in specimens from patients with acute and chronic pancreatitis.

The analysis of 160 sera was performed using two independently developed ELISA systems. To further substantiate the results, all sera were analysed by Western blot technique using a cell lysate of PancTu-1 cells (p53 mutation at codon 176) as the antigen source. Additionally, all positive sera were analysed by Western blot technique using recombinant p53 as the antigen source. We found p53 antibodies in the sera of 5/78 pancreatic tumour patients and in the sera 2/82 of patients with benign pancreatic diseases. Two other specimens from this group tested positive, one with only weak reactivity and the other derived from a patient suffering from a squamous epithelial carcinoma of the tongue in addition to having chronic pancreatitis.

Correspondence: H. Kalthoff.
Received 25 May 1994; and in revised form 12 July 1994.

### Materials and methods

### *Patient groups*

Serum samples from 78 patients with pancreatic cancer were analysed. In 60 patients a tissue diagnosis was obtained. In 57 patients with a malignant tumour the diagnosis was histologically proven adenocarcinoma; three patients had endocrine tumours. Tissue samples were not available in some patients (18) with inoperable cancer who underwent either palliative surgery or endoscopic drainage.

Serum samples of 82 patients with benign pancreatic diseases were analysed in this study. Of these 37 had chronic pancreatitis. The diagnosis of chronic pancreatitis was based on the presence of calcifications, pseudocysts, stenosis or destruction of the pancreatic duct at endoscopic retrograde cholangiopancreatography (ERCP). In 27 patients the pancreatitis was not a first-time event, but no gross structural changes of the pancreas were recorded in these cases. The diagnosis was based on typical clinical signs such as case history, symptoms, elevation of amylase and lipase. Eighteen of the patients had an acute pancreatitis and no medical history of previous pancreatic diseases.

### *ELISA systems*

An anti-p53 autoantibody sandwich ELISA with solid-phase recombinant p53 protein was purchased from Dianova (Hamburg, Germany) and the analyses of the sera performed according to the manufacturer's recommendations. All sera were analysed in a second ELISA system kindly provided by T. Soussi, Paris (Schlichtholz *et al.*, 1994).

### *Cell line and culture conditions*

The human pancreatic cell line PancTu-1 (Dr M. v. Bülow, Mainz, Germany) was routinely cultured in RPMI-1640

medium, supplemented with 10% fetal bovine serum (FBS), 2 mM glutamine, 1 mM sodium pyruvate, 100 U $ml^{-1}$ penicillin and 100 U $ml^{-1}$ streptomycin (all from Life Technologies, Eggenstein, Germany). The cells were grown to approximately 70–90% confluence at 37°C with 5% carbon dioxide.

*Solubilisation*

Cells were washed once with ice-cold phosphate-buffered saline (PBS). Subsequently, cells were washed twice with cell wash buffer [ice-cold PBS, containing 1.5 mM EDTA, 100 μM phenyl methyl sulphonyl fluoride (PMSF), 1 μg $ml^{-1}$ aprotinin (Trasylol), adjusted to pH 7.4] and then resuspended in cell wash buffer. The cell suspension was centrifuged for 4 min at 1,400 r.p.m. and the cell pellet resuspended in lysis buffer [10 mM TRIS, containing 1.5 mM EDTA, 100 μM PMSF, 1 μg $ml^{-1}$ aprotinin (Trasylol) adjusted to pH 7.4]. The cell suspension was incubated for 5 min at 4°C with occasional gentle mixing, followed by the addition of 20 μl of AEA (Antigen Extraction Agent, Oncogene Science, Dianova) for every 100 μl of cell suspension, with further incubation for 5 min at 4°C and occasional vortexing. The extracts were transferred to microcentrifuge tubes and centrifuged at 4°C for 15 min at 14,000 r.p.m. The supernatant was collected and retained for protein determination and Western blotting.

The protein content of the cellular extracts was measured by the BCA Protein Assay (Pierce, Rockford, IL, USA).

*Western blot*

PancTu-1 cell lysate and recombinant wt p53 protein, kindly provided by W. Deppert (HPI, Hamburg, Germany), were electrophoretically separated by SDS–PAGE in a 10% gel and subsequently transferred to nitrocellulose filters.

After blotting, the nitrocellulose sheets were blocked in 5% bovine serum albumin (BSA) in PBS for 3 h at room temperature. The sheets were washed three times for 5 min with washing buffer (PBS, 0.05% Tween 20). Strips were incubated with the primary antibodies for 2 h and 30 min at room temperature on a rocking platform. As positive controls we used MAb 1801 (Oncogene Science, Dianova) and PAb HSP 53/2 (IgG fraction of a rabbit, hyperimmunised with recombinant wt p53 protein). All serum samples were diluted 1:100 in PBS containing 5% BSA. Following the first incubation, the strips were washed three times for 5 min with washing buffer (PBS, 0.05% Tween 20). As secondary antibodies – corresponding to the first antibodies – we used a peroxidase-conjugated AffiniPure $F(ab')_2$ fragment of goat anti-human IgA + IgG + IgM (H + L), a peroxidase-conjugated AffiniPure $F(ab')_2$ fragment of goat anti-mouse (H + L) and a peroxidase-conjugated AffiniPure goat anti-rabbit IgG $F(ab')_2$ fragment (all from Jackson Immunoresearch Laboratories, USA), which were diluted 1:1,000 in PBS containing 5% BSA. Incubation was performed on a rocking platform for 1 h at room temperature. The subsequent washing steps were performed as described above. To visualise the reaction, the strips were incubated in a reagent comprising 4 ml of 4-chloro-1-naphthol [0.3% (w/v) in methanol] and 20 ml of substrate buffer (20 mM) Tris pH 7.4, 150 mM sodium chloride) and 6 μl of hydrogen peroxide (30%, v/v). The reaction was stopped with distilled water.

### Results

In both the independently developed ELISA systems, 5 of 78 serum samples from patients with pancreatic cancer were positive for anti-p53 antibodies (Table Ia). Specimens which were positive by the ELISA methods were confirmed by the Western blot techniques using PancTu-1 cell lysate (Figure 1) and recombinant wt p53 protein (Figure 2) as antigen sources. In all five patients the tumour had metastasised into the liver. In two of the patients a histological tissue diagnosis was available and showed an adenocarcinoma. Four of the patients were males. The age ranged from 45 to 72 years with a median of 59.4 years. All five patients had high CA 19/9 levels and three patients had high CEA levels. All five patients died within 7 months after the pancreatic cancer was diagnosed (the survival time ranging from 2 to 7 months).

Seventy-three serum samples from patients with pancreatic cancer were negative in both ELISA systems and confirmed by Western blot technique. In 67 of the 73 p53 antibody-negative patients with pancreatic cancer the tumours had metastasised into the liver as well. In 58 of the patients a tissue diagnosis was obtained fifty-five were classified as adenocarcinomas and three as endocrine tumours. Fifty-two

**Table I** Detection of anti-p53 antibodies in human sera

| *Sera* | *Assay 1* | *Assay 2* | *PancTu WB* | *wt p53 WB* | *CEA (ng $ml^{-1}$)* | *CA 19/9 (U $ml^{-1}$)* |
|---|---|---|---|---|---|---|
| (a) *Pancreatic carcinoma* | | | | | | |
| 1254 | ++ | ++ | ++ | ND | 21.7 | 23,114 |
| 2437 | + | + | + | + | 2 | 6,300 |
| 2804 | + | ++ | ++ | ++ | 2,443 | 20,570 |
| 3622 | ++ | +++ | +++ | +++ | 5 | 141 |
| 3632 | + | + | + | + | 42 | 5,760 |
| (b) *Benign pancreatic diseases* | | | | | | |
| 2,360 | + | – | + | ++ | ND | ND |
| 2,722 | + | + | + | ++ | 2.7 | 338 |
| 1,134 | + | – | – | +– | ND | ND |
| 1,154 | + | – | – | + | ND | ND |
| 1,033[a] | + | ++ | + | + | ND | 11 |

(a) Results of the serum sample analysis of five patients with malignant pancreatic diseases which were considered positive. Serum samples from 73 patients with negative results are not listed.

(b) Results of the serum sample analysis of two patients with benign pancreatic diseases which were considered positive and two further samples from one patient (1,134 and 1,154) which scored differently in the various assays.

[a]This sample was obtained from a patient suffering from squamous epithelial carcinoma of the tongue in addition to chronic pancreatitis. Serum samples from 78 patients with negative results are not listed.

In assay 1 (Dianova) positivity was evaluated as recommended by the manufacturer yielding the following categories: +, low; ++, medium; +++, high expression. Assay 2 (Th. Soussi, Paris): +, dil. 1:100; ++, dil. 1:300; +++, dil. 1:800. Western blot (WB) analysis: +, low; ++, clear; +++, strong signal; ND, not done.

**Figure 1** Western blot analyses of serum samples (1:100) using PancTu-1 cell lysate as the antigen source. As a positive control MAb 1801 and PAb HSP 53/2 (IgG fraction of a rabbit, hyperimmunised with recombinant wt p53 protein) were used. 'control' means negative control, omitting the first antibody. Sera 457 and 1,419 represent negative specimens. Sera 2,804 and 2,811 are samples from the same patient collected independently at different times (sera 3,622 and 3,627 likewise). These parallel samples served as internal references.

**Figure 2** Western blot analyses of serum samples (1:100) using recombinant wt p53 protein as the antigen source. MAb 1801 served as a positive control, as a negative control human serum 3,522 was used.

patients were males. The age ranged from 40 to 77 years with a median of 60.4 years. Sixty-seven patients had either high CEA or high CA 19/9 levels or both (data not shown).

In the group of patients with benign pancreatic disease, one patient with chronic and one with actue pancreatitis were positive for p53 antibodies (Table 1b). One patient (serum no. 2,360) with acute pancreatitis and no previous history of pancreatic disease tested positive in the ELISA system from Dianova, but not in the other ELISA system. Both Western blot techniques showed a clear reaction (Figures 1 and 2). At the time of serum sample collection this patient was suspected of having autoimmune hepatitis. One patient (serum no. 2,722) with acute pancreatitis, a prior history of pancreatitis and no signs of malignancy on ERCP or endoscopic ultrasonography, and who died from septic shock 1 month after admission to hospital, was positive in both ELISA systems (Table Ib). A reaction was seen with the Western blot technique using PancTu-1 cell lysate as the antigen source and was confirmed by a strong signal in the Western blot with the recombinant wt p53 (Figures 1 and 2). One patient (serum no. 1,033) with a chronic pancreatitis was positive in both ELISA systems and showed strong reactions in both Western blot systems (Table Ib, Figures 1 and 2). This patient had a squamous epithelial carcinoma of the tongue at the time of sample collection. Since the squamous epithelial carcinoma as well as the chronic pancreatitis could have been the reason for the anti-p53 positivity the patient's data are listed separately in Table Ib.

In addition to the three patients described above, one patient (sera nos. 1,134 and 1,154) had a chronic pancreatitis and no malignancy or any other disease at the time of serum collection. A resection of the pancreatic head (partial duodenopancreatectomie) was performed 6 months later and histology only revealed characteristics of an advanced chronic pancreatitis. No signs of malignancy were recorded. The serum samples were positive in the Dianova ELISA system, but showed no clear reaction in the other ELISA system. No reaction was seen with the Western blot technique using the PancTu-1 cell lysate, but a faint reaction was seen with the recombinant p53 (Table Ib, Figures 1 and 2).

### Discussion

The results of the two independently developed ELISA systems for the detection of antibodies against p53 correlated well, both with each other and with the Western blot analysis using PancTu-1 cell lysate and recombinant wt p53 as the antigen source.

Although the rate of p53 mutations in pancreatic tumours is of the order of that in other adenocarcinomas ($\geqslant 50\%$), only a few cases of antibody response were found in this study. Antibodies against p53 were detected in 5/78 patients (6.4%) with pancreatic cancer. The detection rate for antibodies against p53 was reported to be 9–14% in breast cancer (Crawford *et al.*, 1982; Caron de Freomentel *et al.*, 1987; Davidoff *et al.*, 1992; Schlichtholz *et al.*, 1992), 10% in lung cancer (Winter *et al.*, 1992), 12.5% in colorectal cancer (Crawford *et al.*, 1984), 20% in B-cell lymphoma (Caron de Fromentel *et al.*, 1987) and 20% in hepatocellular carcinoma (Volkmann *et al.*, 1993).

In an analysis of 790 serum samples from patients with various malignancies, only 16 positive samples (2%) were identified by Hassapoglidou and Diamandis (1992). The prevalence of p53 antibodies in the patients with pancreatic cancer in our study is lower than in serum samples of patients with colorectal cancer (Crawford *et al.*, 1984), despite the fact that the rate of p53 gene mutations in colorectal and pancreatic cancer is very similar, suggesting either a particular mutation pattern in pancreatic cancer with low immunogenicity or a generally suppressed immune system in these patients. Complexes between p53 protein and a 70 kDa heat shock protein might be necessary for the antigenic presentation of p53 (Davidoff *et al.*, 1992). This putative prerequisite may only be fulfilled in a few pancreatic carcinomas.

In previous analyses (Kalthoff *et al.*, 1993; Kessler *et al.*, 1993) we were able to show specific p53 immunoreactivity in cytospin preparations derived from patients with pancreatic cancer (7/10) and, in addition, in specimens from patients with acute and chronic pancreatitis (9/13). The fact that antibodies against p53 were found in serum samples of patients with non-malignant pancreatic diseases may point to a p53 release and antigen processing with subsequent elicitation of a p53-directed antibody response by necroinflammatory benign diseases. Another hypothesis to explain the occurrence of p53 antibodies in these patients is the presence of as yet undetected malignancies.

We are grateful to Professor Thierry Soussi, Paris, for kindly providing his ELISA system and to Professor Wolfgang Deppert, Hamburg, for kindly providing recombinant wt p53 protein. We acknowledge the excellent technical assistance of Britta Büsing. We thank Alan Ladak, London, for critical reading of the manuscript. This study was supported by the E. u. G. Roggenbuck Stiftung, Hamburg, Germany. The results are part of the thesis of J. Marxsen.

### References

BARTON, C.M., STADDON, S.L., HUGHES, C.M., HALL, P.A., O'SULLIVAN, C., KLOPPEL, G., THEIS, B., RUSSELL, R.C., NEOPTOLEMOS, J., WILLIAMSON, R.C.N., LANE, D.P. & LEMOINE, N.R. (1991). Abnormalities of the p53 tumour suppressor gene in human pancreatic cancer. *Br. J. Cancer*, **64**, 1076–1082.

CARON DE FROMENTEL, C. & SOUSSI, T. (1992). TP53 tumor suppressor gene: a model for investigating human mutagenesis. *Genes Chrom. Cancer*, **4**, 1–15.

CARON DE FROMENTEL, C., MAY-LEVIN, F., MOURIESSE, H., LEMERLE, J., CHANDRASEKARAN, K. & MAY, P. (1987). Presence of circulating antibodies against cellular protein p53 in a notable proportion of children with B-cell lymphoma. *Int. J. Cancer*, **39**, 185–189.

CASEY, G., YAMANAKA, Y., FRIESS, H., KOBRIN, M.S., LOPEZ, M.E., BUCHLER, M., BEGER, H.G. & KORC, M. (1993). p53 Mutations are common in pancreatic cancer and are absent in chronic pancreatitis. *Cancer Lett.*, **69**, 151–160.

CRAWFORD, L.V., PIM, D.C. & BULBROOK, R.D. (1982). Detection of antibodies against the cellular protein p53 in sera from patients with breast cancer. *Int. J. Cancer*, **30**, 403–408.

CRAWFORD, L.V., PIM, D.C. & LAMB, P. (1984). The cellular protein p53 in human tumors. *Mol. Biol. Med.*, **2**, 261–272.

DAVIDOFF, A.M., IGLEHART, J.D. & MARKS, J.R. (1992). Immune response to p53 is dependent upon p53/HSP70 complexes in breast cancers. *Proc. Natl Acad. Sci. USA*, **89**, 3439–3442.

HARRIS, C.C. & HOLLSTEIN, M. (1993). Clinical implications of the p53 tumor-suppressor gene. *N. Engl. J. Med.*, **329**, 1318–1327.

HASSAPOGLIDOU, S. & DIAMANDIS, E.P. (1992). Antibodies to the p53 tumor suppressor gene product quantified in cancer patient serum with a time-resolved immunofluorometric technique. *Clin. Biochem.*, **25**, 445–449.

HOLLSTEIN, M., SIDRANSKY, D., VOGELSTEIN, B. & HARRIS, C.C. (1991). p53 mutations in human cancers. *Science*, **253**, 49–53.

KALTHOFF, H., SCHMIEGEL, W., ROEDER, C., KASCHE, K., SCHMIDT, A., LAUER, G., THIELE, H.-G., HONOLD, G., PANTEL, K., RIETHMÜLLER, G., SCHERER, E., MAURER, J. MAACKE, H. & DEPPERT, W. (1993). p53 and K-Ras alterations in pancreatic epithelial cell lesions. *Oncogene*, **8**, 289–298.

KESSLER, A., SCHMIEGEL, W., RÖDER, C., SOEHENDRA, N. & KALTHOFF, H. (1993). Diagnostische Bedeutung von anti-p53-Antikörpern in der Pankreaszytopathologie. *Z. Gastroenterol.*, **31**, 541.

LANE, D.P. (1993). A death in the life of p53. *Cancer*, **362**, 786–787.

LEVINE, A.J., MOMAND, J. & FINLAY, C.A. (1991). The p53 tumour suppressor gene. *Nature*, **351**, 453–456.

LEVINE, A.J., PERRY, M.E., CHANG, A., SILVER, A., DITTMER, D., WU, M. & WELSH, D. (1994). The 1993 Walter Hubert Lecture: the role of the p53 tumour-suppressor gene in tumorigenesis. *Br. J. Cancer*, **69**, 409–416.

RUGGERI, B., ZHANG, S.Y., CAAMANO, J., DIRADO, M., FLYNN, S.D. & KLEIN, S.A. (1992). Human pancreatic carcinomas and cell lines reveal frequent and multiple alterations in the p53 and Rb-1 tumor-suppressor genes. *Oncogene*, **7**, 1503–1511.

SCARPA, A., CAPELLI, P., MUKAI, K., ZAMBONI, G., ODA, T., IACANO, C. & HIROHASHI, S. (1993). Pancreatic adenocarcinomas frequently show p53 gene mutations. *Am. J. Pathol.*, **142**, 1534–1543.

SCHLICHTHOLZ, B., LEGROS, Y., GILLET, D., GAILLARD, C., MARTY, M., LANE, D., CALVO, F. & SOUSSI, T. (1992). The immune response to p53 in breast cancer patients is directed against immunodominant epitopes unrelated to the mutational hot spot. *Cancer Res.*, **52**, 6380–6384.

SCHLICHTHOLZ, B., TREDANIEL, J., LUBIN, R., ZALCMAN, G., HIRSCH, A. & SOUSSI, T. (1994). Analyses of p53 antibodies in sera of patients with lung carcinoma define immunodominant regions in the p53 protein. *Br. J. Cancer*, **69**, 809–816.

VOGELSTEIN, B. & KINZLER, K.W. (1992). p53 function and dysfunction. *Cell*, **70**, 523–526.

VOLKMANN, M., MULLER, M., HOFMANN, W.J., MEYER, M., HAGELSTEIN, J., RATH, U., KOMMERELL, B., ZENTGRAF, H. & GALLE, P.R. (1993). The humoral immune response to p53 in patients with hepatocellular carcinoma is specific for malignancy and independent of the alpha-fetoprotein status. *Hepatology*, **18**, 559–565.

WINTER, S.F., MINNA, J.D., JOHNSON, B.E., TAKAHASHI, T., GAZDAR, A.F. & CARBONE, D.P. (1992). Development of antibodies against p53 in lung cancer patients appears to be dependent on the type of p53 mutation. *Cancer Res.*, **52**, 4168–4174.